# Identifying children exposed to maltreatment: a systematic review update

**DOI:** 10.1186/s12887-020-2015-4

**Published:** 2020-03-07

**Authors:** Jill R. McTavish, Andrea Gonzalez, Nancy Santesso, Jennifer C. D. MacGregor, Chris McKee, Harriet L. MacMillan

**Affiliations:** 1 Department of Psychiatry & Behavioural Neurosciences, McMaster University, 1280 Main Street West, MIP 201A, Hamilton, ON L8S 4K1 Canada; 2 Department of Health Research Methods, Evidence, and Impact, McMaster University, 1280 Main Street West, 2C Area, Hamilton, ON L8S 4K1 Canada; 3 Faculty of Information & Media Studies, Western University, FIMS & Nursing Building, Room 2050, London, ON N6A 5B9 Canada; 4 Department of Pediatrics, McMaster University, 1280 Main Street West, MIP 201A, Hamilton, ON L8S 4K1 Canada

**Keywords:** Child maltreatment, Screening, Case-finding, Identification

## Abstract

**Background:**

Child maltreatment affects a significant number of children globally. Strategies have been developed to identify children suspected of having been exposed to maltreatment with the aim of reducing further maltreatment and impairment. This systematic review evaluates the accuracy of strategies for identifying children exposed to maltreatment.

**Methods:**

We conducted a systematic search of seven databases: Medline, Embase, PsycINFO, Cumulative Index to Nursing and Allied Health Literature, Cochrane Libraries, Sociological Abstracts and the Education Resources Information Center. We included studies published from 1961 to July 2, 2019 estimating the accuracy of instruments for identifying potential maltreatment of children, including neglect, physical abuse, emotional abuse, and sexual abuse. We extracted data about accuracy and narratively synthesised the evidence. For five studies—where the population and setting matched known prevalence estimates in an emergency department setting—we calculated false positives and negatives. We assessed risk of bias using QUADAS-2.

**Results:**

We included 32 articles (representing 31 studies) that evaluated various identification strategies, including three screening tools (SPUTOVAMO checklist, Escape instrument, and a 6-item screening questionnaire for child sex trafficking). No studies evaluated the effects of identification strategies on important outcomes for children. All studies were rated as having serious risk of bias (often because of verification bias). The findings suggest that use of the SPUTOVAMO and Escape screening tools at the population level (per 100,000) would result in hundreds of children being missed and thousands of children being over identified.

**Conclusions:**

There is low to very low certainty evidence that the use of screening tools may result in high numbers of children being falsely suspected or missed. These harms may outweigh the potential benefits of using such tools in practice (PROSPERO 2016:CRD42016039659).

## Background

Child maltreatment, including physical abuse, sexual abuse, emotional abuse, and neglect impacts a significant number of children worldwide [1–3]. For example, a survey involving a nationally representative sample of American children selected using telephone numbers from 2013 to 2014 found that lifetime rates of maltreatment for children aged 14 to 17 was 18.1% for physical abuse, 23.9% for emotional abuse, 18.4% for neglect, and 14.3% and 6.0% for sexual abuse of girls and boys respectively [4]. Child maltreatment is associated with many physical, emotional, and relationship consequences across the lifespan, such as developmental delay first seen in infancy; anxiety and mood disorder symptoms and poor peer relationships first seen in childhood; substance use and other risky behaviours often first seen in adolescence; and increased risk for personality and psychiatric disorders, relationship problems, and maltreatment of one’s own children in adulthood [5–9]. Given the high prevalence and serious potential negative consequences of child maltreatment, clinicians need to be informed about strategies to accurately identify children potentially exposed to maltreatment, a task that “can be one of the most challenging and difficult responsibilities for the pediatrician” [10]. Two main strategies for identification of maltreatment—screening and case-finding—are often compared to one another in the literature [11, 12]. Screening involves administering a standard set of questions, or applying a standard set of criteria, to assess for the suspicion of child maltreatment in all presenting children (“mass screening”) or high-risk groups of children (“selective screening”). Case-finding, alternatively, involves providers being alert to the signs and symptoms of child maltreatment and assessing for potential maltreatment exposure in a way that is tailored to the unique circumstances of the child.

A previous systematic review by Bailhache et al. [13] summarized “evidence on the accuracy of instruments for identifying abused children during any stage of child maltreatment evolution before their death, and to assess if any might be adapted to screening, that is if accurate screening instruments were available.” The authors reviewed 13 studies addressing the identification of physical abuse (7 studies), sexual abuse (4 studies), emotional abuse (1 study), and multiple forms of child maltreatment (1 study). The authors noted in their discussion that the tools were not suitable for screening, as they either identified children too late (i.e., children were already suffering from serious consequences of maltreatment) or the performance of the tests was not adaptable to screening, due to low sensitivity and specificity of the tools [13].

This review builds upon the work of Bailhache et al. [13] and performs a systematic review with the objective of assessing evidence about the accuracy of instruments for identifying children suspected of having been exposed to maltreatment (neglect, as well as physical, sexual abuse, emotional abuse). Similar to the review by Bailhache et al. [13], we investigate both screening tools and other identification tools or strategies that could be adapted into screening tools. In addition to reviewing the sensitivity and specificity of instruments, as was done by Bailhache et al. [13], for five studies, we have also calculated estimates of false positives and negatives per 100 children, a calculation which can assist providers in making decisions about the use of an instrument [14]. This review contributes to an important policy debate about the benefits and limitations of using standardized tools (versus case-finding) to identify children exposed to maltreatment. This debate has become increasingly salient with the publication of screening tools for adverse childhood experiences, or tools that address child maltreatment alongside other adverse experiences [15, 16].

It should be noted here that while “screening” typically implies identifying *health problems*, screening for child maltreatment is different in that it usually involves identifying *risk factors* or *high-risk groups.* As such, while studies evaluating tools that assist with identification of child maltreatment are typically referred to as diagnostic accuracy studies [17], the word “diagnosis” is potentially misleading. Instead, screening tools for child maltreatment typically codify several risk and clinical indicators of child maltreatment (e.g., caregiver delay in seeking medical attention without adequate explanation). As such, they may more correctly be referred to as tools that identify potential maltreatment, or signs, symptoms and risk factors that have a strong association with maltreatment and may lead providers to consider maltreatment as one possible explanation for the sign, symptom, or risk factor. Assessment by a health care provider should then include consideration of whether there is reason to suspect child maltreatment. If maltreatment is suspected, this would lead to a report to child protection services (CPS) in jurisdictions with mandatory reporting obligations (e.g., Canada, United States) or to child social services for those jurisdictions bound by occupational policy documents (e.g., United Kingdom) [18]. Confirmation or verification of maltreatment would then occur through an investigation by CPS or a local authority; they, in turn, may seek consultation from one or more health care providers with specific expertise in child maltreatment. Therefore, throughout this review we will refer to identification tools as those that aid in the identification of *potential* child maltreatment.

## Methods

A protocol for this review is registered with the online systematic review register, PROSPERO (PROSPERO 2016:CRD42016039659) and study results are reported according to the Preferred Reporting Items for Systematic Reviews and Meta-Analyses (PRISMA) checklist (see supplemental file 1). As the review by Bailhache et al. [13] considered any English or French materials published between 1961 and April 2012 (only English-language materials were retrieved from their search), we searched for English-language materials published between 2012 and July 2, 2019 (when the search was conducted). Additional inclusion criteria are found in Table 1. Inclusion criteria for this review were matched to those in Bailhache et al.’s [13] review. We included diagnostic accuracy studies [17] that 1) evaluated a group of children by a test, examination or other procedure (hereafter referred to as the index test) designed to identify children potentially exposed to maltreatment and also 2) evaluated the same group of children (or ideally a random subsample) by a reference standard (acceptable reference standards are listed in Table 1) that confirmed or denied exposure to potential maltreatment. We excluded articles that assessed psychometric properties of child maltreatment measures unless diagnostic data was available in the paper.

**Table 1:** Inclusion and exclusion criteria

**Inclusion criteria** 1. **Population.** Children (0 < 18) 2. **Intervention (index test).** Any instrument that assessed if children had been exposed to physical abuse, sexual abuse, emotional abuse, or neglect. Studies had to describe the index test with enough information to be replicable. 3. **Comparator (reference test).** Studies had to have an acceptable reference standard, i.e. “expert assessments, such as child’s court disposition; substantiation by the child protection services or other social services; assessment by a medical, social or judicial team using one or several information sources (caregivers or child interview, child symptoms, child physical examination, and other medical record review)” [13]. 4. **Outcomes.** Studies had to assess one of the following outcomes: sensitivity, specificity, positive predictive value, or negative predictive value. 5. **Study design.** Studies need not include a comparison population (e.g., case series could be included if the intention was to evaluate one of the outcomes listed above).	
**Exclusion criteria** 1. **Ineligible population.** Studies that only addressed adults’ or children’s exposure to intimate partner violence. 2. **Ineligible intervention (index test).** Studies that identified a clinical indicator for child maltreatment, such as retinal hemorrhaging, but not child maltreatment itself and tools that identified a different population (e.g., general failure to thrive, children’s exposure to intimate partner violence). 3. **Ineligible comparator (reference test).** Studies that did not have an acceptable reference standard (e.g., parent reports of abuse were ineligible). 4. **Ineligible outcomes.** Studies that at minimum did not set out to evaluate at least one of the following accuracy outcomes: sensitivity, specificity, positive predictive value, negative predictive value. 5. **Ineligible publication types.** Studies published as abstracts were excluded, as not enough information was available to critically appraise the study design. Also excluded were studies published in non-article format, such as books or theses. The latter were excluded for pragmatic issues, but recent research suggests that inclusion of these materials may have little impact on results [19].	

The searches for the review update were conducted in seven databases: Medline, Embase, PsycINFO, Cumulative Index to Nursing and Allied Health Literature, Sociological Abstracts, the Education Resources Information Center, and Cochrane Libraries (see supplemental file 2 for example search). Forward and backward citation chaining was also conducted to complement the search. All articles identified by our searches were screened independently by two reviewers at the title and abstract and full-text level. An article suggested for inclusion by one screener was sufficient to forward it to full-text review. Any disagreements at full text stage were resolved by discussion.

### Data extraction and analysis

For all included studies, one author extracted the following data: study design, the study’s inclusion criteria, form of potential child maltreatment assessed, index tool, sample size, reference standard, and values corresponding to sensitivity and specificity. While our original protocol indicated that we would extract and analyze data about child outcomes (e.g., satisfaction, well-being), service outcomes (e.g., referral rates), and child well-being outcomes (e.g., internalizing symptoms, externalizing symptoms, suicidal ideation) from the studies (e.g., from randomized trials that evaluated screening versus another identification strategy and assessed associated outcomes), no such data were available. Extracted data were verified by a second author by cross-checking the results in all tables with data from the original articles. Disagreements were resolved by discussion.

Sensitivity and specificity are “often misinterpreted and may not reflect well the effects expected in the population of interest” [14]. Other accuracy measures, such as false positives and false negatives, can be more helpful for making decisions about the use of an instrument [14], but determining them requires a reasonable estimate of prevalence in the intended sample (in this case of the exposure, child maltreatment) and in the intended setting (e.g., emergency department). Although there are no clear cut-off points for acceptable proportions of false negatives and positives, as acceptable cut-offs depend on the clinical setting and patient-specific factors, linking false positives and negatives to downstream consequences (e.g., proportion of children who will undergo a CPS investigation who should not or who miss being investigated) can assist practitioners in determining acceptable cut-offs for their practice setting.

For those studies where prevalence estimates were available, sensitivity and specificity values were entered into GRADEpro software in order to calculate true/false positives/negatives per 100 children tested. This free, online software allows users to calculate true/false positives/negatives when users enter sensitivity and specificity values of the index test and an estimate of prevalence. In GRADEpro, true/false positives/negatives can be calculated across 100, 1000, 100,000, or 1,000,000 patients. We selected 100 patients as a total, as it allows easy conversion to percentage of children. We also give an example of true/false positives/negatives per 100,000 children tested, which is closer to a population estimate or numbers across several large, emergency departments. To calculate these values, two prevalence rates were used (2 and 10%) based on the range of prevalence of child maltreatment in emergency departments in three high-income country settings [20], as most of the identified screening tools addressed children in these settings. Use of these prevalence rates allow for a consistent comparison of true/false positives/negatives per 100 children across all applicable studies. For consistency and to enhance accuracy of calculations in GRADEpro of true/false positives/negatives proportions per 100, where possible, all sensitivity and specificity values and confidence intervals for the included studies were recalculated to six decimal places (calculations for confidence intervals used: p ± 1.96 × √p(1-p)/n]). In GRADEpro, the formula for false positives is (1 - specificity)*(1 - prevalence) and the formula for false negatives is (1 - sensitivity)*(prevalence). As the majority of studies differed in either a) included populations or b) applied index tests, we were unable to pool data statistically across the studies. Instead, we narratively synthesized the results by highlighting the similarities and differences in false positives/negatives across the included studies.

For the population estimate, we modeled the effects of the SPUTOVAMO checklist for children with physical abuse or neglect on downstream consequences for children under 8 years of age presenting to the emergency department with any physical injury. We calculated true/false positives/negatives per 100,000 using the lower end of the prevalence range (2%) [20]. Based on American estimates, we assumed that 17% of children who are reported to child welfare are considered to have substantiated maltreatment and among children with substantiated maltreatment, 62% may receive post-investigation services [21]. We also modeled downstream consequences of false negatives, based on an estimate that 25 to 50% of children who are exposed to maltreatment need services for mental health symptoms [22]. We modeled consequences of false positives by assuming that all suspicions lead to reports which lead to CPS investigations.

### Critical appraisal

One author critically appraised each study using the QUADAS-2 tool [23] and all data were checked by a second author, with differences resolved through consensus. The QUADAS-2 tool evaluates risk of bias related to a) patient selection, b) index test, c) reference standard, and d) flow and timing. Questions related to “applicability” in QUADAS-2 were not answered because they overlap with questions involved in the GRADE process [17]. As the developers of QUADAS-2 note [23], an overall rating of “low” risk of bias is only possible when all domains are assessed as low risk of bias. An answer of “no” to any of the questions indicates that both the domain (e.g., “patient selection”) and the overall risk of bias for the study is high. In this review, a study was rated as “high” risk of bias if one or more domains was ranked as high risk of bias, a study was ranked as “low” risk of bias when all domains were rated as low risk of bias and a study was ranked as “unclear” risk of bias otherwise (i.e., when the study had one or more domains ranked as “unclear” risk of bias and no domains ranked as “high” risk of bias).

### Grading of recommendations, assessment, development and evaluation (GRADE)

Evidence was assessed using GRADE [17]. GRADE rates our certainty that the effect we present is close to the true effect; the certainty that the effect we present is close to the true effect is rated as high, moderate, low or very low certainty. A GRADE rating is based on an assessment of five domains: (1) risk of bias (limitations in study designs); (2) inconsistency (heterogeneity) in the direction and/or size of the estimates of effect; (3) indirectness of the body of evidence to the populations, interventions, comparators and/or outcomes of interest; (4) imprecision of results (few participants/events/observations, wide confidence intervals); and (5) indications of reporting or publication bias. For studies evaluating identification tools and strategies, a body of evidence starting off with cross-sectional accuracy studies is considered “high” certainty and then is rated down to moderate, low, or very low certainty based on the five factors listed above.

## Results

**Fig. 1:**
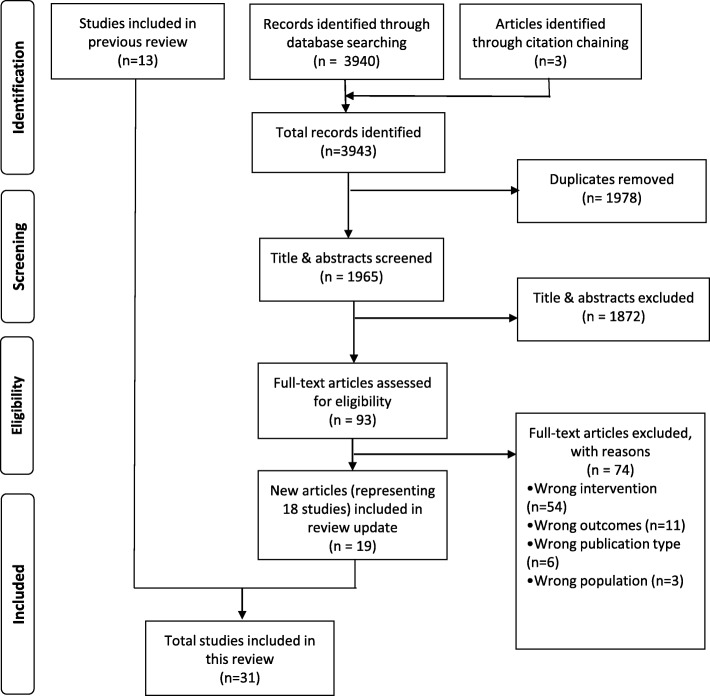
PRISMA Flow Diagram

The updated search and citation chaining retrieved 3943 records; after de-duplication, 1965 titles and abstracts were screened for inclusion (see Fig. 1). From this set of results, 93 full-text articles were reviewed for inclusion, of which 19 new articles (representing 18 studies) were included [24–42]. In addition, the 13 studies evaluated in the Bailhache et al. review [43–55] were included in this review update, for a total of 32 articles (31 studies).

### Study characteristics

Overall, we did not find any studies that measured important health outcomes after the use of a screening tool or other instrument. Instead, the included tools and strategies provided accuracy estimates for a range of maltreatment types (see supplemental file 3 for study characteristics), including multiple types of maltreatment (6 studies); medical child maltreatment (also known as caregiver fabricated illness in a child, factitious disorder imposed on another and Munchausen syndrome by proxy, 1 study); sexual abuse (7 studies), including child sex trafficking (3 studies); emotional abuse (1 study); and physical abuse (18 studies), including abusive head trauma (11 studies).

### Risk of bias and GRADE assessment of included studies

One study was rated as having an unclear risk of bias and all remaining studies were rated as high risk of bias, with 23 studies (72%) having high risk of bias across two or more domains (see supplemental file 4 for critical appraisal rankings). A number of studies used very narrow age ranges to test their index test, representing potentially inappropriate exclusions for the basis of studying identification strategies. For example, while very young children (under 5 years of age) are at most risk of serious impairment and death from physical abuse including abusive head trauma, rates of non-fatal physical abuse peak between 3 and 12 years [56]. Ideally, index tests that seek to identify potential physical abuse should address all children who are legally entitled to protection (or at a minimum, address children ≤12 years).

A number of studies did not apply the reference standard to all children and instead only applied it to a subset of children who were positively identified by the index test or some other method, which can lead to serious verification bias (i.e., no data for the number of potentially maltreated children missed). For example, the reference standard was applied to only 55/18275 (0.3%) of the children in the study by Louwers et al. [26]. Only Sittig et al. [27], in a study assessing one of the recently published screening tests, applied the reference standard to a random sample of 15% of the children who received a negative screen by the index test, thereby reducing the potential for serious verification bias. A few studies also used the index test as part of the reference standard, which can lead to serious incorporation bias. For example, Greenbaum et al. [37] noted that the 6-item child sex trafficking screening questions were “embedded within the 17-item questionnaire,” which was used by the reference standard (health care providers) to determine if child sex trafficking potentially occurred.

Using the GRADE approach to evaluate the certainty of evidence, the included studies started at high certainty as all but six studies were cross-sectional studies. The evidence was rated down due to very serious concerns for risk of bias (making the evidence “low” certainty) and further rated down for imprecision (making the evidence “very low” certainty).

### General accuracy

Table 2 reports sensitivity and specificity rates for each study. Studies are organized according to child maltreatment type (multiple types of maltreatment, medical child maltreatment, sexual abuse, child sex trafficking, emotional abuse, physical abuse and neglect, and abusive head trauma). The type of child maltreatment assessed by each tool is specified, as is the name of the identification strategy.

**Table 2:** Accuracy outcomes for each child maltreatment identification tool with 95% confidence intervals (CI)

Study ID, Country	Form of child maltreatment	Index assessment	Sample size	Sensitivity (95% CI)^**a**^	Specificity (95% CI)^**a**^
**Child maltreatment (multiple types)**					
Teeuw 2019, The Netherlands [42]	Neglect, exposure to intimate partner violence, physical, sexual, emotional abuse, pediatric condition falsification	SPUTOVAMO checklist	193	78 (67–86)^b^	36 (27–45)^b^
		Top-to-toe inspection	131	53 (39–66)^b^	54 (42–65)^b^
		SPUTOVAMO checklist and top-to-toe inspection	124	78 (64–88)^b^	30 (20–42)^b^
Schouten 2017, The Netherlands [24]	Neglect, exposure to intimate partner violence,physical, emotional, and sexual abuse	SPUTOVAMO-R2 checklist	5592	15 (7–27)	98 (98–99)
Dinpanah 2017, Iran	Neglect, exposure to intimate partner violence, physical, emotional, and sexual abuse	Escape tool	6120	100 (88–100)	98 (98–99)
Louwers 2014, The Netherlands [26]	Neglect, exposure to intimate partner violence, physical, emotional, and sexual abuse	Escape tool	18,275	80 (67–89)	98 (98–99)
Bernstein 1997, United States [44]	Sexual abuse	Childhood trauma questionnaire	213	86 (73–94)	76 (69–82)
	Physical abuse		227	82 (71–89)	73 (65–80)
	Emotional abuse		229	79 (69–87)	72 (64–79)
	Physical neglect		274	77 (63–87)	61 (54–67)
***Medical child maltreatment***					
Greiner 2013, United States [31]	Medical child maltreatment	Medical child abuse instrument using a cutoff score ≥ 4	408	95 (72–100)	96 (93–97)
**Sexual abuse**					
Cheung 2004, China [46]	Sexual abuse	Colposcopic examination of anal and genital findings	77	56 (31–79)	98 (90–100)
Berenson 2002, United States [43]	Sexual abuse	A horizontal hymen diameter of ≥6.5 mm in knee chest position	327	29 (22–37)	86 (80–90)
Drach 2001, United States	Sexual abuse	Child sexual behavior inventory (CSBI)	209	50 (37–63)^b^	50 (41–58)^b^
Wells 1997, United States [55]	Sexual abuse	Abbreviated structured interview for symptoms associated with sexual abuse (ASASA)	74	91 (69–98)^b^	88 (76–95)^b^
***Child sex trafficking***					
Kaltiso, 2018, United States [39]	Child sex trafficking	Six-item screening questionnaire for child sex trafficking	203	91 (57–100)	53 (46–60)
Greenbaum 2018a, United States [36]	Child sex trafficking	Same as above	810	84 (75–91)	60 (56–64)^b^
Greenbaum 2018b, United States [37]	Child sex trafficking	Same as above	108	92 (72–99)	73 (62–82)
**Emotional abuse**					
Fernandopulle 2003, Sri Lanka [48]	Emotional abuse	Scale for identifying emotional abuse	98	77 (56–90)	51 (39–63)
**Physical abuse and neglect**					
Kemp 2018, United Kingdom and Ireland [40]	Physical abuse and neglect—Burns (scalds)	Burns Risk Assessment for Neglect or Abuse Tool – validation study	373	88 (60–98)	82 (77–85)
	Physical abuse and neglect—Burns (non-scalds)		372	82 (65–93)	79 (74–83)
	Physical abuse and neglect—Burns (scalds)	Burns Risk Assessment for Neglect or Abuse Tool – derivation study	768	71 (58–82)	73 (70–76)
	Physical abuse and neglect—Burns (non-scalds)		559	66 (52–78)	75 (71–79)
Berger 2018, United States [35]	Physical abuse	Triggers in electronic medical record	10,936	97 (88–99)	98 (98–99)^b^
Sittig 2016, The Netherlands [27]	Physical abuse	SPUTOVAMO checklist	720	100 (31–100)	86 (84–89)
	Neglect		720	83 (35–100)	87 (84–89)
Bousema 2016, The Netherlands [28]	Physical abuse	SPUTOVAMO checklist	442	73 (57–86)	95 (92–96)
Pierce 2010, United States [50]	Physical abuse	Bruising classification and regression tree	71	97 (82–100)	84 (68–93)
Valvano 2009, United States [51]	Physical abuse	Bruising associated with a fracture	150	26 (18–36)	75 (62–85)
Chang 2005, United States [45]	Physical abuse	Screening index for physical child abuse (SIPCA)	58,558	87 (83–90)	80 (80–81)
***Abusive head trauma***					
Hymel 2019 [38] Companion papers: Hymel 2013, 2014 [33, 34]	Abusive head trauma	Seven-variable clinical predication rule	474	87 (83–91)	90 (85–93)
Pfeiffer 2018 [41]	Abusive head trauma	Pediatric Brain Injury Research Network clinical prediction rule	122	96 (80–100)	43 (33–53)
Palifka 2016, United States [29]	Abusive head trauma	Lacerations	165	13 (8–20)	100 (85–100)
Cowley 2015, United Kingdom & France [30]	Abusive head trauma	Predicting Abusive Head Trauma tool	198	72 (60–82)	86 (78–91)
Acker 2014, United States [32]	Abusive head trauma	Hematocrit (proportion of blood in red blood cells) ≤30% on presentation	921	48 (43–53)^b^	81 (77–84)^b^
Hymel 2014, United States [33] Companion paper: Hymel 2019 [38]	Abusive head trauma	Four-variable clinical prediction rule	291	96 (90–99)	43 (35–50)
Hymel 2013 [34], United States Companion paper: Hymel 2019 [38]	Abusive head trauma	Five-variable abusive head trauma clinical prediction rule	209	96 (89–99)	36 (27–46)
Vinchon 2010, France [52]	Abusive head trauma	Brain ischemia	84	27 (15–42)^b^	97 (85–100)^b^
		Subdural hematoma		82 (67–91)^b^	56 (40–72)^b^
		Severe retinal hemorrhage		56 (40–70)^b^	97 (85–100)^b^
		Absence of scalp swelling		98 (87–100)	77 (60–88)^b^
Vinchon 2005, France [53]	Abusive head trauma	Retinal hemorrhage grade 1,2 or 3	129	75 (61–85)	93 (84–97)
		Retinal hemorrhage grade 2 or 3		66 (52–78)	100 (94–100)
Hettler 2003, United States [49]	Abusive head trauma	No history of trauma	163	69 (54–81)	97 (92–99)
Wells 2002, United States [54]	Abusive head trauma	Four variable model for predicting AHT	257	84 (77–89)	83 (74–89)

^a^CIs for sensitivity and specificity rates were calculated using the following formula: p ± 1.96 × √p(1-p)/n

^b^Sensitivity and specificity values were recalculated from provided true positive and true negative values

In addition to the studies previously reviewed by Bailhache et al. [13], this systematic review update identified three screening tools, as well as an identification tool for medical child maltreatment, “triggers” embedded in an electronic medical record, four clinical prediction tools, and two predictive symptoms of abusive head trauma. False positive/negative values are reported only for the studies using screening tools with samples where the prevalence of child maltreatment could be estimated; all values for the studies identified in the Bailhache et al. [13] review are available in Table 2.

#### Screening instruments

Three screening instruments were identified in this systematic review update: 1) the SPUTOVAMO checklist, 2) the Escape instrument, and 3) a 6-item screening questionnaire for child sex trafficking. The SPUTOVAMO checklist [24, 27, 28, 42] is a screening instrument that determines whether there is a suspicion of child maltreatment via a positive answer to one or more of five questions (e.g., injury compatible with history and corresponding with age of child?). Its use is mandatory in Dutch emergency departments and “out-of-hours” primary care locations. Two studies [24, 42] evaluated if the SPUTOVAMO checklist could detect potential physical abuse, sexual abuse, emotional abuse, neglect, or exposure to intimate partner violence in children under 18 years of age presenting to either out-of-hours primary care locations [24] or an emergency department [42] in the Netherlands. Two separate studies reported on the use of the SPUTOVAMO checklist to assess for potential exposure to physical abuse in children under 8 years of age presenting to the emergency department with a physical injury [27] or children under 18 years of age presenting to a burn centre with burn injuries [28].

Two studies evaluated the Escape instrument [25, 26], a screening instrument very similar in content and structure to the SPUTOVAMO checklist. The Escape instrument involves five questions (e.g., is the history consistent?) that are used to assess for potential physical abuse, sexual abuse, emotional abuse, neglect, and exposure to intimate partner violence in children under 16 years of age [25] or 18 years of age [26] presenting to an emergency department.

Three studies [36, 37, 39] reported on use of a 6-item screening questionnaire for child sex trafficking, where an answer to two or more questions (e.g., Has the youth ever run away from home?) indicated suspicion of a child being exposed to sex trafficking. The studies tested the screening questionnaire in children of a similar age group (10,11, or 12 to 18 years of age) presenting to emergency departments [36, 37, 39], child advocacy centres or teen clinics [37].

Five studies [24–27, 42] had samples where the prevalence of child maltreatment could be estimated. In other words, each study’s included sample was similar enough (e.g., children less than 18 years presenting to the emergency department) to match 2% to 10% prevalence estimates found in emergency departments [20]. As shown in Table 3, the Sittig et al. [27] study, which evaluated the SPUTOVAMO checklist, found that per 100 children tested, 0 potentially physically abused children were missed and 0 to 2 potentially neglected children were missed. Twelve to 13 children were falsely identified as potentially physically abused or neglected.

**Table 3:** False positives and negatives for screening studies with 95% confidence intervals (CI)

Study ID, Country	Sample Size	Per 100			
		False negatives (95% CI)	False positives (95% CI)	False Negatives (95% CI)	False positives (95% CI)
		2% prevalence		10% prevalence	
**Child maltreatment (multiple types)**					
Teeuw 2019, The Netherlands [42]	193	0 (0–1)	63 (54–71)	2 (1–3)	58 (49–66)
	131	1 (1–1)	45 (34–57)	5 (3–6)	42 (31–52)
	124	0 (0–1)	69 (57–78)	2 (1–4)	63 (53–72)
Schouten 2017, The Netherlands [24]	5592	2 (1–2)	2 (1–2)	9 (7–9)	2 (1–2)
Dinpanah 2017, Iran [25]	6120	0 (0–0)	2 (1–2)	0 (0–1)	2 (1–2)
Louwers 2014, The Netherlands [26]	18,275	0 (0–1)	2 (2–2)	2 (1–3)	2 (2–2)
**Physical abuse and neglect**					
Sittig 2016, The Netherlands [27]	720	0 (0–1)	13 (11–16)	0 (0–7)	12 (10–15)
	720	0 (0–1)	13 (11–16)	2 (0–6)	12 (10–14)

The other studies suffered from verification or incorporation bias leading to a sensitivity estimate that is too high (underestimating false negative estimates) and a specificity estimate that is too high (underestimating false positive estimates). These studies [24–26, 42] found that per 100 children tested, 0 to 9 potentially maltreated children were missed and 2 to 69 children were falsely identified as potentially maltreated. For the studies that evaluated the SPUTOVAMO checklist specifically [24, 42], 0 to 9 potentially maltreated children were missed and 2 to 69 children were falsely identified as potentially maltreated. For the studies that evaluated the Escape tool [25, 26], 0 to 2 children were missed and 2 children were falsely identified as potentially maltreated.

#### Modelling service outcomes of the SPUTOVAMO checklist for physical abuse or neglect based on a population estimate

After using a screening tool, children will receive some type of service depending on the results. We modelled what would happen to children after the use of the SPUTOVAMO checklist on a population level per 100,000 children (see supplemental file 5 for modelling using the Escape instrument).

When using the SPUTOVAMO checklist, providers may correctly identify 2000 children potentially exposed to physical abuse and 1666 potentially exposed to neglect. American estimates [21] suggest 17% of children who are reported to child welfare are substantiated and 62% of substantiated children receive post-investigation services. Using these estimates, this means that some form of post-investigative services may be received by 211 children with substantiated physical abuse and 176 children with substantiated neglect.

No children exposed to potential physical abuse and 334 children who have been exposed to potential neglect would be missed. Since an estimated 25 to 50% of children who are exposed to maltreatment need services for mental health symptoms [21], 84 children potentially exposed to neglect would not be referred for the mental health services they need.

In addition, we calculated that 13,230 children would be misidentified as potentially physically abused and 13,034 children would be misidentified as potentially neglected. Although these children would likely receive an assessment by a qualified physician that would determine they had not experienced maltreatment, all of these children could undergo a stressful and unwarranted child protection services investigation.

#### Medical child maltreatment instrument

Greiner et al. [31] evaluated a “medical child maltreatment” instrument (also known as caregiver fabricated illness in a child [57] or factitious disorder imposed on another [58]), where a positive answer to four or more of the 15 questions indicated suspicion of medical child maltreatment (e.g., caregiver has features of Munchausen syndrome (multiple diagnoses, surgeries, and hospitalizations, with no specific diagnosis)).

#### Triggers in an electronic medical record

Berger et al. [35] evaluated “triggers” added to an electronic medical record to help identify children under 2 years of age at risk for physical abuse (e.g., a “yes” response to “Is there concern for abuse or neglect?” in the pre-arrival documentation by a nurse; documentation of “assault” or “SCAN” as the chief complaint). This study suffers from serious verification bias, since only abused children and a small, non-random sample (*n* = 210) were evaluated by the reference standard.

#### Clinical predication rules and predictive symptoms

Five studies (published in six articles) evaluated four clinical prediction tools (Burns Risk Assessment for Neglect or Abuse Tool, Pediatric Brain Injury Research Network clinical prediction rule, Predicting Abusive Head Trauma, and Hymel’s 4- or 5- or 7-variable prediction models).

Kemp et al. [40] investigated the Burns Risk Assessment for Neglect or Abuse Tool, a clinical prediction rule to assist with the recognition of suspected maltreatment, especially physical abuse or neglect. Hymel et al. evaluated a five-variable clinical prediction rule (derivation study) [34] and a four-variable clinical prediction rule (validation study) [33] in identifying potential abusive head trauma in children less than 3 years of age who were admitted to the post-intensive care unit for management of intracranial injuries. An additional article by Hymel et al. [38] combined the study populations in the derivation and validation studies in order to evaluate a seven-variable clinical prediction rule in identifying potential abusive head trauma. The seven-variable clinical prediction rule used seven indicators to predict potential abusive head trauma (e.g., any clinically significant respiratory compromise at the scene of injury, during transport, in the emergency department, or prior to admission).

Pfeiffer et al. [41] evaluated the Pediatric Brain Injury Research Network clinical prediction rule. This clinical prediction rule evaluated the likelihood of abusive head trauma in acutely head-injured children under 3 years of age admitted to the post-intensive care unit. The authors recommended that children who presented with one or more of the following four predictor variables should be evaluated for abuse (respiratory compromise before admission; any bruising involving ears, neck, and torso; any subdural hemorrhages and/or fluid collections that are bilateral or interhemispheric; any skull fractures other than an isolated, unilateral, nondiastatic, linear parietal skull fracture).

Two studies evaluated different predictive symptoms of abusive head trauma (parenchymal brain lacerations and hematocrit levels ≤30% on presentation). Palifika et al. [29] examined the frequency of lacerations in children less than 3 years of age who had abusive head trauma (as determined by the institutional child abuse team) compared with accidentally injured children with moderate-to-severe traumatic brain injury. For children under 5 years of age who were admitted to one of two level-one pediatric trauma centres with a diagnosis of traumatic brain injury, Acker et al. [32] identified hematocrit values of 30% or less as a finding that should prompt further investigation for potential abusive head trauma.

## Discussion

This review updates and expands upon the systematic review published by Bailhache et al. [13] and was conducted to evaluate the effectiveness of strategies for identifying potential child maltreatment. Since the publication of Bailhache et al.’s [13] systematic review, there have been 18 additional studies published. The included studies reported the sensitivity and specificity of three screening tools (the SPUTOVAMO checklist, the Escape instrument, and a 6-item screening questionnaire for child sex trafficking), as well as the accuracy of an identification tool for medical child maltreatment, “triggers” embedded in an electronic medical record, four clinical prediction tools (Burns Risk Assessment for Neglect or Abuse Tool, Pediatric Brain Injury Research Network clinical prediction rule, Predicting Abusive Head Trauma, and Hymel’s 4- or 5- or 7-variable prediction models), and two predictive symptoms of abusive head trauma (parenchymal brain lacerations and hematocrit levels ≤30% on presentation). As the Bailhache et al. [13] systematic review identified no screening tools, the creation of the SPUTOVAMO checklist, Escape instrument, and 6-item child sex trafficking screening questionnaire represents a notable development since their publication. The recent creation of an identification tool for child sex trafficking also reflects current efforts to recognize and respond effectively to this increasingly prevalent exposure. Aside from these new developments, many of the other points discussed by Bailhache et al. [13] were confirmed in this update: it is still difficult to assess the accuracy of instruments to identify potential child maltreatment as there is no gold standard for identifying child maltreatment; what constitutes “maltreatment” still varies somewhat, as does the behaviours that are considered abusive or neglectful (e.g., we have excluded children’s exposure to intimate partner violence, which is increasingly considered a type of maltreatment); and it is still challenging to identify children early in the evolution of maltreatment (many of the identification tools discussed in this review are not intended to identify children early and as such children are already experiencing significant consequences of maltreatment).

The studies included in this systematic review provide additional evidence that allow us to assess the effectiveness of strategies for identifying potential exposure to maltreatment. Based on the findings of this review (corresponding with the findings of Bailhache et al.’s [13] review), we found low certainty evidence and high numbers of false positives and negatives when instruments are used to screen for potential child maltreatment. Although no studies assessed the effect of screening tools on child well-being outcomes or recurrence rates, based on data about reporting and response rates [21, 22], we can posit that children who are falsely identified as potentially maltreated by screening tools will likely receive a CPS investigation that could be distressing. Furthermore, maltreated children who are missed by screening tools will not receive or will have delayed access to the mental health services they need.

We identified several published instruments that are not intended for use as screening tools, such as clinical prediction rules for abusive head trauma. Clinical prediction tools or rules, such as Hymel’s variable prediction model, combine medical signs, symptoms, and other factors in order to predict diseases or exposures. While they may be useful for guiding clinicians’ decision-making, and may be more accurate than clinical judgement alone [59], they are not intended for use as screening tools. Instead, the tools “act as aids or prompts to clinicians to seek further clinical, social or forensic information and move towards a multidisciplinary child protection assessment should more information in support of AHT [abusive head trauma] arise” [41]. As all identification tools demand clinician time and energy, widespread implementation of any (or a) clinical prediction tool is not warranted until it has undergone three stages of testing: derivation (identifying factors that have predictive power), validation (demonstrating evidence of reproducible accuracy), and impact analysis (evidence that the clinical prediction tool changes clinician behaviour and improves patient important outcomes) [60]. Similar to the findings of a recent systematic review on clinical prediction rules for abusive head trauma [41], in this review we did not find any clinical prediction rules that had undertaken an impact analysis. However, several recent studies have considered the impact of case identification via clinical prediction rules. This includes assessing if the Predicting Abusive Head Trauma clinical prediction rule alters clinicians’ abusive head trauma probability estimates [61], emergency clinicians’ experience with using the Burns Risk Assessment for Neglect or Abuse Tool in an emergency department setting [62], and cost estimates for identification using the Pediatric Brain Injury Research Network clinical predication rule as compared to assessment as usual [63]. Additional research on these clinical predication rules may determine if such rules are more accurate than a clinician’s intuitive estimation of risk factors for potential maltreatment or how the tool impacts patient-important outcomes.

Many of the included studies had limitations in their designs, which lowered our confidence in their reported accuracy parameters. Limitations in this area are not uncommon. A recent systematic review by Saini et al. [64] assessed the methodological quality of studies assessing child abuse measurement instruments (primarily studies assessing psychometric properties). The authors found that “no instrument had adequate levels of evidence for all criteria, and no criteria were met by all instruments” [64]. Our review also resulted in similar findings to the original review by Bailhache et al. [13], in that 1) most studies did not report sufficient information to judge all criteria in the risk of bias tool; 2) most studies did not clearly blind the analysis of the reference standard from the index test (or the reverse); 3) some studies [26, 36, 37, 39] included the index test as part of the reference standard (incorporation bias), which can overestimate the accuracy of the index test; and 4) some studies used a case-control design [29, 31, 36], which can overestimate the performance of the index test. A particular challenge, also noted by Bailhache et al. [13], was the quality of reporting in many of the included studies. Many articles failed to include clear contingency tables in reporting their results, making it challenging for readers to fully appreciate missing values and potentially inflated sensitivity and specificity rates. For example, one study evaluating the SPUTOVAMO checklist reported 7988 completed SPUTOVAMO checklists. However, only a fraction of these completed checklists were evaluated by the reference standard (verification bias, discussed further below) (193/7988, 2.4%) and another reference standard (a local CPS agency) was used to evaluate an additional portion of SPUTOVAMO checklists (246/7988, 3.1%). However, the negative predictive and positive predictive value calculations were based on different confirmed cases. Ideally missing data and indeterminate values should be reported [23]. Researchers have increasingly called for diagnostic accuracy studies to report indeterminate results as sensitivity analysis [65].

Verification bias was a particular study design challenge in the screening studies identified in this review. For example, Dinpanah et al. [25] examined the accuracy of the Escape instrument, a five-question screener applied in emergency department settings, for identifying children potentially exposed to physical abuse, sexual abuse, emotional abuse, neglect, or intimate partner violence. The authors report a sensitivity and specificity of 100 and 98 respectively. While the accuracy was high, their study suffered from serious verification bias as approximately 137 out of 6120 (2.2%) of children suspected of having been maltreated received the reference standard. For the children who did not receive the reference standard, there is no way to ascertain the number of children who were potentially maltreated, but unidentified (false negatives). Furthermore, as inclusion in this study involved a convenience sample of children/families who a) gave consent for participation and b) cooperated in filling out the questionnaire, we do not know if the children in this study were representative of their study population. In addition, unlike screening tools for intimate partner violence [66, 67], none of the screening for possible maltreatment tools have been evaluated through randomized controlled trials; as such, we have no evidence about the effectiveness of such tools on reducing recurrence of maltreatment or improving child well-being.

This review identified one study which evaluated a screening tool that did not suffer from serious verification bias or incorporation bias. Sittig et al. [27] evaluated the ability of the SPUTOVAMO five-question checklist to identify potential physical abuse or neglect in children under the age of 8 years who presented to an emergency department with any physical injury. While no children exposed to potential physical abuse were missed by this tool, at a population level a large number of children were falsely identified as potentially physically abused (over 13,000); furthermore, at a population level, many children potentially exposed to neglect were missed by this tool (334 per 100,000). Qualitative research suggests that physicians report having an easier time detecting maltreatment based on physical indicators, such as bruises and broken bones, but have more challenges identifying less overt forms of maltreatment, such as ‘mild’ physical abuse, emotional abuse, and children’s exposure to intimate partner violence [68]. The authors of this study suggest that the SPUTOVAMO “checklist is not sufficiently accurate and should not replace skilled assessment by a clinician” [27].

The poor performance of screening tests for identifying children potentially exposed to maltreatment that we found in this review leads to a similar conclusion to that reached for the World Health Organization’s Mental Health Gap Action Programme (mhGAP) update, which states that “there is no evidence to support universal screening or routine inquiry” [69]. Based on the evidence, the mhGAP update recommends that, instead of screening, health care providers use a case-finding approach to identify children exposed to maltreatment by being “alert to the clinical features associated with child maltreatment and associated risk factors and assess for child maltreatment, without putting the child at increased risk” [69]. As outlined in the National Institute for Health and Clinical Excellence (NICE) guidance for identifying child maltreatment, indicators of possible child maltreatment include signs and symptoms; behavioural and emotional indicators or cues from the child or caregiver; and evidence-based risk factors that prompt a provider to consider, suspect or exclude child maltreatment as a possible explanation for the child’s presentation [70]. The NICE guidance includes a full set of maltreatment indicators that have been determined based on the results of their systematic reviews [70]. This guidance also discusses how providers can move from “considering” maltreatment as one possible explanation for the indicator to “suspecting” maltreatment, which in many jurisdictions invokes a clinician’s mandatory reporting duty. In addition, there are a number of safety concerns that clinicians must consider before inquiring about maltreatment, such as ensuring that when those children who are of an age and developmental stage where asking about exposure to maltreatment is feasible, this should occur separately from their caregivers and that systems for referrals are in place [71].

The findings of this review have important policy and practice implications especially since, as noted in the introduction, there is an increasing push to use adverse childhood experiences screening tools in practice [15, 16]. While we are not aware of any diagnostic accuracy studies evaluating adverse childhood experiences screening tools, it is unclear how these tools are being used in practice, or how they will in the future be used in practice [72]. For example, does a provider who learns a child has experienced maltreatment via an adverse childhood experiences screener then inform CPS authorities? What services is the child entitled to based on the findings of an adverse childhood experiences screener, if the child indicates they have experienced child maltreatment along with other adverse experiences? The findings of the present review suggest that additional research is needed on various child maltreatment identification tools (further accuracy studies, along with studies that assess acceptability, cost effectiveness, and feasibility) before they are implemented in practice. The findings also suggest the need for more high-quality research about child maltreatment identification strategies, including well-conducted cohort studies that follow a sample of children identified as not maltreated (to reduce verification bias) and randomized controlled trials that assess important outcomes (e.g., recurrence and child well-being outcomes) in screened versus non-screened groups. The results of randomized controlled trials that have evaluated screening in adults experiencing intimate partner violence underscore the need to examine the impacts of screening [66, 67]. Similar trials in a child population could help clarify risks and benefits of screening for maltreatment. Future systematic reviews that assess the accuracy of tools that attempt to identify children exposed to maltreatment by evaluating parental risk factors (e.g., parental substance use) would also complement the findings of this review.

### Strengths and limitations

The strengths of this review include the use of a systematic search to capture identification tools, the use of an established study appraisal checklist, calculations of false positives and negatives per 100 where prevalence estimates were available (which may be more useful for making clinical decisions than sensitivity and specificity rates), and the use of GRADE to evaluate the certainty of the overall evidence base. A limitation is that we included English-language studies only. There are limitations to the evidence base, as studies were rated as unclear or high risk of bias and the overall certainty of the evidence was low. Additional limitations include our reliance on 2 and 10% prevalence rates commonly seen in emergency departments [20] and our use of American estimates to model potential service outcomes following a positive screen (e.g., number of children post-investigation who receive services). These prevalence rates likely do not apply across different countries where prevalence rates are unknown. For example, one study evaluated the Escape instrument in an Iranian emergency department. While the authors cite the 2 to 10% prevalence rate in their discussion [25], we are unaware of any studies estimating prevalence of child maltreatment in Iranian emergency departments. When known, practitioners are encouraged to use the formulas in the methods section (or to use GRADEpro) to estimate false positives and negatives based on the prevalence rates of their setting, as well as known estimates for service responses in their country, in order to make informed decisions about the use of various identification strategies. Furthermore, our modelling of services outcomes assumes that 1) all positives screens will be reported and 2) that reports are necessarily stressful/negative. While many of the included studies that used CPS as a reference standard reported all positive screens, it is unclear if this would be common practice outside of a study setting (i.e., does a positive screen trigger one’s reporting obligation?). Further research is needed to determine likely outcomes of positive screens. It is also important to recognize that while reviews of qualitative research do identify that caregivers and mandated reporters have negative experiences and perceptions of mandatory reporting (and associated outcomes), there are some instances where reports are viewed positively by both groups [68, 73]. Finally, because our review followed the inclusion/exclusion criteria of Bailhache et al. [13] and excluded studies that did not explicitly set out to evaluate sensitivity, specificity, positive predictive values or negative predictive values, it is possible that there are additional studies where such information could be calculated.

## Conclusion

There is low to very low certainty evidence that the use of screening tools may result in high numbers of children being falsely suspected or missed. These harms may outweigh the potential benefits of using such tools in practice. In addition, before considering screening tools in clinical programs and settings, research is needed that identifies patient-important outcomes of screening strategies (e.g., reduction of recurrence).

## Supplementary information

**Additional file 1.** PRISMA Checklist

**Additional file 2.** Example search strategy

**Additional file 3.** Study and participant characteristics of interest

**Additional file 4.** Critical appraisal rankings

**Additional file 5.** Consequences of screening per 100,000 children

## Data Availability

All data is available within this article, supplemental material or via the references.

## Abbreviations

CPS (Child Protective Services): A short form for governmental agencies responsible for providing child protection, including responses to reports of maltreatment
GRADE (Grading of Recommendations, Assessment, Development and Evaluation): The GRADE process involves assessing the certainty of the best available evidence and is often used to support guideline development processes
mhGAP (Mental Health Gap Action Programme): A program launched by the World Health Organization’s Mental Health Gap Action Programme, in order to facilitate the scaling up of care for mental, neurological, and substance use disorders; the program is comprised of evidence-based guidelines and practical intervention guides used to assist in the implementation of guideline principles
NICE (National Institute for Health and Care Excellence): An executive non-departmental body operating in the United Kingdom that provides national guidance and advice to improve health and social care
QUADAS-2 (Quality Assessment of Studies of Diagnostic Accuracy-2): A tool for evaluating the quality of diagnostic accuracy studies
