# Supplementary material for: Identifying children exposed to maltreatment: a systematic review update
Source: BMC Pediatr. 2020 Mar 7;20:113. doi: 10.1186/s12887-020-2015-4 (PMC7060650; doi:10.1186/s12887-020-2015-4)
Supplement: Supplementary file 2 — Additional file 2. Example search strategy [file 12887_2020_2015_MOESM2_ESM.docx]

**SUPPLEMENTAL FILE 2 – Example search strategy**

Database: Ovid MEDLINE(R) Epub Ahead of Print, In-Process & Other Non-Indexed Citations, Ovid MEDLINE(R) Daily and Ovid MEDLINE(R) <1946 to Present>
Search Strategy:
--------------------------------------------------------------------------------
1     exp Child Abuse/ or shaken baby syndrome/ or incest/ (28035)
2     ((child* or girl? or boy? or infant* or baby or babies or toddler* or preschool* or pre-school* or teen* or adolescen* or youth*) adj3 (abuse? or abusing or maltreat* or neglect* or assault* or rape? or raping or molest* or (sex* adj2 abus*))).tw,kw. (24062)
3     or/1-2 (36961)
4     exp child abuse/di (4859)
5     (detect* or diagnos* or screen* or identify* or identification or case-find*).tw,kw. (4898115)
6     or/4-5 (4901217)
7     3 and 6 (11586)
8     "Reproducibility of Results"/ (329941)
9     "Sensitivity and Specificity"/ (311687)
10     "Predictive Value of Tests"/ (170146)
11     sensitivity.tw,kw. (649113)
12     specificity.tw,kw. (384520)
13     validat*.tw,kw. (366513)
14     predict*.tw,kw. (1215574)
15     or/8-14 (2560605)
16     7 and 15 (2019)
17     limit 16 to (english language and yr="2012 -Current") (644)

**Search updated to July 2, 2019:**

Database: Ovid MEDLINE(R) Epub Ahead of Print, In-Process & Other Non-Indexed Citations, Ovid MEDLINE(R) Daily and Ovid MEDLINE(R) <1946 to Present>
Search Strategy:
--------------------------------------------------------------------------------
1     exp Child Abuse/ or shaken baby syndrome/ or incest/ (30265)
2     ((child* or girl? or boy? or infant* or baby or babies or toddler* or preschool* or pre-school* or teen* or adolescen* or youth*) adj3 (abuse? or abusing or maltreat* or neglect* or assault* or rape? or raping or molest* or (sex* adj2 abus*))).tw,kw. (27366)
3     or/1-2 (41024)
4     exp child abuse/di (5259)
5     (detect* or diagnos* or screen* or identify* or identification or case-find*).tw,kw. (5634762)
6     or/4-5 (5638054)
7     3 and 6 (13200)
8     "Reproducibility of Results"/ (377993)
9     "Sensitivity and Specificity"/ (337008)
10     "Predictive Value of Tests"/ (192020)
11     sensitivity.tw,kw. (745927)
12     specificity.tw,kw. (435151)
13     validat*.tw,kw. (465461)
14     predict*.tw,kw. (1449688)
15     or/8-14 (2988527)
16     7 and 15 (2421)
17     limit 16 to (english language and yr="2012 -Current") (414)
