# Supplementary material for: Identifying children exposed to maltreatment: a systematic review update
Source: BMC Pediatr. 2020 Mar 7;20:113. doi: 10.1186/s12887-020-2015-4 (PMC7060650; doi:10.1186/s12887-020-2015-4)
Supplement: Supplementary file 3 — Additional file 3. Study and participant characteristics of interest [file 12887_2020_2015_MOESM3_ESM.docx]

| **SUPPLEMENTAL FILE 3 – Study and participant characteristics of interest** | | | | | |
| --- | --- | --- | --- | --- | --- |
| **Study ID, Country** | **Inclusion criteria** | **Form of child maltreatment** | **Index assessment** | **Baseline sample size** | **Reference standard** |
| **Child maltreatment** | | | | | |
| Teeuw 2019, The Netherlands | Children <18 years presenting to the emergency department | Neglect, exposure to IPV, physical, sexual, emotional abuse, pediatric condition falsification | SPUTOVAMO checklist and/or complete physical examination (top-to-toe inspection) | 12 198 | A final Expert Panel diagnosis was made of all the children who were tested positive by the Child Abuse and Neglect Team (TASK) |
| Schouten 2017,  The  Netherlands | Children <18 years presenting to Out-of-hours primary care locations | Neglect, exposure to intimate partner violence, physical, sexual, and emotional abuse | SPUTOVAMO-R2 checklist | 5592 | Report to child protection services in 10 months following attendance at the out-of-hours primary care locations |
| Dinpanah 2017,  Iran | Children <16 years of age who presented to emergency department | Neglect, exposure to intimate partner violence, physical, sexual, and emotional abuse | Escape tool | 6120 | Reference standard was a child abuse team (pediatrician, social worker, forensic physician) who only examined children who screened positive for potentially experiencing child abuse (who were identified by a trained emergency medicine specialist) |
| Louwers 2014,  The Netherlands | Children <18 years of age who visited one of three emergency departments | Neglect, exposure to intimate partner violence, physical, sexual, and emotional abuse | Escape tool | 18,275 | Only suspected cases were independently evaluated by an expert panel consisting of four physicians with extensive experience in child abuse, of whom one was a forensic pediatrician and two were pediatricians |
| Bernstein 1997,  United States | Adolescents (12-17) who were admitted to a private psychiatric hospital | Neglect, exposure to intimate partner violence, physical, sexual, and emotional abuse | Childhood trauma questionnaire | 398 | Therapist assessment using admission notes, discharge summary, and patient’s hospital chart |
| **Medical child maltreatment** | | | | | |
| Greiner 2013, United States | Children <18 years with confirmed medical child abuse, and children <18 who were hospitalized with  the discharge diagnosis of apnea, diarrhea/ vomiting, or seizures/epilepsy | Medical child maltreatment | Medical child abuse screening instrument using a cutoff score ≥4 | 408 | All patients included in the confirmed medical child maltreatment cohort were previously reported to CPS and medical child maltreatment had been substantiated; all controls had no concerns for medical child maltreatment in their records and none had been reported to CPS |
| **Sexual abuse** | | | | | |
| Cheung 2004,  China | Girls <17 referred to sexual abuse evaluation team at hospital | Sexual abuse | Colposcopic examination of anal and genital findings | 77 | Assessment by multidisciplinary team involved in assessing CPS investigation. Team used medical history, behavioural changes, laboratory results, and anogenital findings |
| Berenson 2002,  United States | Girls 3-8 years of age, presenting at pediatric clinic with and without sexual abuse | Sexual abuse | A horizontal hymen  diameter of ≥6.5 mm in knee chest position | 386 | Review of medical record, completion of Child Sexual Behaviour Inventory by parent, interview with child in private by nurse, psychologist, or social worker |
| Drach 2001, United States | Children between 2-12 years referred to child abuse program for sexual abuse allegations | Sexual abuse | Child sexual behavior inventory | 209 | Assessment by multidisciplinary team using forensic medical evaluation, statements made by the child during forensic interviewing, and an assessment of relevant historical information |
| Wells 1997, United States | Boys <16 with confirmed or alleged sexual abuse or no sexual abuse | Sexual abuse | Abbreviated structured interview for symptoms associated with sexual abuse | 121 | Confirmed sexual abuse involved perpetrator confession, alleged sexual abuse involved medical examiner evaluation |
| ***Child sex trafficking*** | | | | | |
| Kaltiso 2018 | Children 10-18 years who were English-speaking and presented to the pediatric emergency department with high-risk chief complaints, which were a priori defined as those potentially associated with child sex trafficking. Patients were also included if the attending physician was concerned about high-risk sexual or social behavior regardless of the chief complaint. | Child sex trafficking | Six-item screening questionnaire for child sex trafficking | 203 | Patient electronic medical records were reviewed to gather additional information that might assist in determining victim status. A patient was considered to be a “true” child sex trafficking victim if any information gathered during the emergency department visit indicated that their circumstances fulfilled the federal definition of child sex trafficking. This included exchange of sex for money, subsistence items or luxury items, exchange of sex for monetary or nonmonetary gain of another individual, involvement in production  of child sexual abuse materials, and performing in sex-oriented businesses. |
| Greenbaum 2018a | Children 11-17 years who were English-speaking who presented to participating emergency departments, child advocacy centres, or teen clinics. Emergency departments maintained an  additional inclusion criterion that the chief patient complaint must be acute sexual assault/abuse, or concern for child sex trafficking. | Child sex trafficking | Six-item screening questionnaire for child sex trafficking | 810 | Healthcare providers involved in the study were asked, “Based on questions from this survey and on any other information gleaned from the visit, do you believe this patient is a victim of commercial sexual exploitation of children/child sex trafficking? (e.g., exchanging a sex act for something of value to child or another person, including survival sex, prostitution, sex trafficking, pornography, working in sex industry) (yes/no/don’t know).” This question was worded to describe the federal definition of child sex trafficking and to ensure, when possible, that the decision about child sex trafficking status was based on all available information from all sources (including the medical chart, information provided by the person accompanying the child, information provided by associated authorities via referral forms, etc.). |
| Greenbaum 2018b | Children 12-18 years who were English-speaking and where there were concerns commercial sexual exploitation, child sex trafficking, or sexual assault/sexual abuse. | Child sex trafficking | Six-item screening questionnaire for child sex trafficking | 108 | Patients were classified as “suspected commercial sexual exploitation or child sex trafficking if the information obtained by the medical provider indicated a high likelihood that the youth had been the victim of such, based on the definitions of the United Nations and the Institute of Medicine. This information may have come from authorities  (e.g., law enforcement reported finding the youth during a raid of a site of known prostitution) or from information gleaned  From the medical record, from parents or others, or from the youth |
| **Emotional abuse** | | | | | |
| Fernandopulle 2003, Sri Lanka | Children 13-15 years in school | Emotional abuse | Scale for identifying emotional abuse | 98 | Psychiatrist assessment during child interview |
| **Physical abuse and neglect** | | | | | |
| Kemp 2018, United Kingdom and Ireland | Children <16 years presenting with a burn to selected pediatric emergency departments, general emergency departments and burns units | Physical abuse, neglect | BuRN-Tool | 787 | Referral to the hospital or Local Authority children’s social care team for investigation of suspected maltreatment. |
| Berger 2018, United States | Children <2 years old who met one of the criteria that activated a trigger embedded into the electronic medical record. | Physical abuse | Child abuse alert system: thirty triggers were embedded into the electronic medical record. | 10,936 | The child protection team assessed for abuse in all reports of suspected maltreatment made by any hospital-based mandated reporter to CPS as well as all trauma-related admissions for children <2 years of age. The child protection team is composed of physicians with expertise in child maltreatment. A nonrandom sample of children who were at high risk of being false negatives was also reviewed by the child protection team. |
| Sittig 2016,  The Netherlands | Children <8 years of age admitted to an emergency room for any physical injury | Physical abuse, neglect | SPUTOVAMO checklist (mandatory implementation) | 720 | Three member expert panel review of 1. Medical file from emergency room visit, 2. Detailed history of injury from child abuse pediatrician, 3. Risk factors derived from general practitioner, youth doctor, and CPS questionnaires, 4. 6-month follow-up information from electronic record |
| Bousema 2016,  The Netherlands | Children <18 years of age presenting with burn injuries to a burn centre | Physical abuse | SPUTOVAMO checklist | 498 | Provision of additional support, as determined by burn centre child abuse team, CPS, or both (i.e., suspected child abuse) |
| Pierce 2010, United States | Children <4 with abusive or accidental trauma | Physical abuse | Bruising classification and regression tree | 95 | Hospital medical team evaluation, CPS evaluation, or stated cause of injury incompatible |
| Valvano 2009, United States | Children <18 with fractures (excluding skull fracture) referred to child abuse team | Physical abuse | Bruising associated with a fracture | 150 | Child abuse experts who reviewed chart summaries of patients |
| Chang 2005, United States | Children <14 years with at least one ICD-9 diagnosis of physical injury | Physical abuse | Screening index for physical child abuse | 58,558 | E codes and certain ICD-9-CM codes in the 995.5x range |
| ***Abusive head trauma*** | | | | | |
| Hymel 2019, United States | Children <3 years of age with an acute head-injury hospitalized for intensive care at one of 18 sites | Abusive head trauma | Seven-variable clinical prediction rule | 500 | A priori definitional criteria: A patient’s head trauma was classified as abusive IF: The primary caregiver admitted abusive acts or abusive acts by the primary caregiver were witnessed by an unbiased, independent observer, or the primary caregiver specifically denied that the pre-ambulatory infant or young child in his/her care had experienced any head trauma or the primary caregiver provided an account of the child’s head injury event that was clearly historically inconsistent with repetition over time or the primary caregiver provided an account of the child’s head injury event that was clearly developmentally inconsistent with the child’s known (or expected) gross motor skills or abuse evaluation revealed patterned bruising or dry contact burns, hot water immersion burns, or CT-confirmed intra-abdominal injury. |
| Pfeiffer 2018, Australia and New Zealand | Children <3 years admitted to pediatric centres with abnormal neuroimaging results | Abusive head trauma | PediBIRN, Four-variable Clinical Prediction Rule | 141 | Abusive head trauma was defined as the diagnosis of cranial or intracranial head injury  (confirmed on neuroimaging), which was due to physical child abuse by parents or caregivers rather than neglect‍ according to the decision of a multidisciplinary child protection  team at the conclusion of their investigation and their consideration of the relevant social, forensic, and clinical features in the context of the presenting history, in accordance with the Australian and New Zealand standard child protection assessment  processes. |
| Palifka 2016, United States | Children <3 years of age with abusive head trauma and children and accidentally injured children with moderate-to severe  traumatic brain injury | Abusive head trauma | Lacerations | 505 | Perpetrator confession and/or evaluation by the institutional child abuse medical evaluation team were determined to have inflicted trauma |
| Cowley 2015, United Kingdom & France | Children <24 months admitted to hospital with an intracranial injury | Abusive head trauma | Predicting abusive head trauma tool | 198 | Thorough multidisciplinary assessment or court proceedings, social and historical factors beyond the presenting injury, or a perpetrator admission or independently witnessed abusive incident |
| Acker 2014, United States | Children ≤5 who were admitted with diagnosis of traumatic brain injury | Abusive head trauma | Hematocrit (proportion of blood in red blood cells) ≤30% on presentation | 921 | Diagnosis of abusive head trauma was made by the child advocacy and protection team, led by a child advocacy expert and includes social workers, nurses, psychologists, psychiatrists and attorneys |
| Hymel 2014, United States | Children <3 admitted to post-intensive care unit for the management of symptomatic, acute, closed, traumatic, cranial, or intracranial injuries confirmed by computed tomography or magnetic resonance imaging | Abusive head trauma | Four-variable clinical prediction rule | 291 | Primary caregiver admission of abusive acts, abusive acts witnessed by an independent observer, primary caregiver denial of head trauma, primary caregiver gives account of injury that is inconsistent or developmentally inconsistent, above or equal to 2 categories of extracranial injuries moderately or high suspicious for abuse |
| Hymel 2013, United States | Children <3 hospitalized acutely in a post-intensive care unit for treatment of symptomatic, acute, closed (nonpenetrating), traumatic, cranial or intracranial injuries confirmed by computed tomography or magnetic resonance imaging | Abusive head trauma | Five-variable abusive head trauma clinical prediction rule | 209 | Same as above |
| Vinchon 2010,  France | Children <2 years referred to the emergency room, the pediatric intensive care unit, or neurosurgical department | Abusive head trauma | Brain ischemia, subdural hematoma, severe retinal hemorrhage, absence of scalp swelling | 84 | Expert assessment by multidisciplinary team (neurosurgeon, pediatrician, forensic doctor, psychologist, and social worker) |
| Vinchon 2005,  France | Children <2 diagnosed with craniocerebral traumatic  injuries diagnosed based on computed tomography scan | Abusive head trauma | Retinal hemorrhage grade 1, 2 or 3 or grade 2 or 3 | 129 | Assessment by multidisciplinary team multidisciplinary team (surgeons, ophthalmologists, pediatricians, forensic  pathologists, and social workers) |
| Hettler 2003, United States | Children <3 admitted to inpatient services with traumatic intracranial hemorrhage | Abusive head trauma | No history of trauma | 163 | Witnessed or confessed abuse, indicators of abusive head trauma (e.g., retinal hemorrhages), CPS notes |
| Wells 2002, United States | Children <3 with intracranial hemorrhage detected via computed tomography scan | Abusive head trauma | Four variable model for predicting abusive head trauma | 293 | Assessment of patient age, sex, circumstances of injury, results of CPS investigations |
