# Supplementary material for: Identifying children exposed to maltreatment: a systematic review update
Source: BMC Pediatr. 2020 Mar 7;20:113. doi: 10.1186/s12887-020-2015-4 (PMC7060650; doi:10.1186/s12887-020-2015-4)
Supplement: Supplementary file 4 — Additional file 4. Critical appraisal rankings [file 12887_2020_2015_MOESM4_ESM.docx]

| **SUPPLEMENTAL FILE 4 – Critical appraisal rankings of low (L), unclear (U), or high (H) bias based on QUADAS-2 questions (Qs).** | | | | | | | | | | | | | | | | |
| --- | --- | --- | --- | --- | --- | --- | --- | --- | --- | --- | --- | --- | --- | --- | --- | --- |
| **Study ID** | **Patient selection bias Qs** | | | | **Index test bias Qs** | | | **Reference standard bias Qs** | | | **Patient flow and timing bias Qs** | | | | | **Risk of Bias** |
|  | **1** | **2** | **3** |  | **4** | **5** |  | **6** | **7** |  | **8** | **9** | **10** | **11** |  |  |
| **Child maltreatment** | | | | | | | | | | | | | | | | |
| Teeuw 2019 | Y | Y | Y | L | Y | - | L | Y | N | U | N | N | N | N | H | High |
| Schouten 2017 | Y | Y | Y | L | N | - | H | N | U | H | U | N | N | Y | H | High |
| Dinpanah 2017 | N | Y | N | H | U | - | U | Y | Y | L | U | N | N | Y | H | High |
| Louwers 2014 | Y | Y | N | H | N | - | H | Y | N | H | U | N | N | N | H | High |
| Bernstein 1997 | U | Y | N | H | N | N | H | Y | Y | L | N | N | N | N | H | High |
| **Sexual abuse** | | | | | | | | | | | | | | | | |
| Cheung 2004 | Y | Y | U | U | U | - | U | U | N | H | U | Y | Y | N | H | High |
| Berenson 2002 | U | N | N | H | U | Y | U | Y | U | U | Y | Y | N | Y | H | High |
| Drach 2001 | U | Y | N | H | U | - | U | Y | Y | L | U | Y | Y | U | U | High |
| Wells 1997 | U | N | U | H | U | N | H | U | U | U | U | Y | N | N | H | High |
| **Child sex trafficking** | | | | | | | | | | | | | | | | |
| Kaltiso 2018 | N | Y | N | H | U | - | U | U | N | U | Y | N | N | Y | H | High |
| Greenbaum 2018a | N | N | N | H | N | N | H | Y | U | U | Y | Y | U | N | U | High |
| Greenbaum 2018b | N | Y | N | H | N | - | U | Y | N | U | Y | Y | Y | N | U | High |
| **Emotional abuse** | | | | | | | | | | | | | | | | |
| Fernandopulle 2003 | Y | Y | U | U | Y | N | H | U | Y | U | Y | Y | Y | U | U | High |
| **Medical child maltreatment** | | | | | | | | | | | | | | | | |
| Greiner 2013 | N | N | N | H | N | N | H | Y | N | H | U | N | N | Y | H | High |
| **PA & N** | | | | | | | | | | | | | | | | |
| Kemp 2018 | Y | Y | Y | L | Y | N | U | N | U | U | Y | N | N | Y | H | High |
| Berger 2018 | Y | Y | N | U | U | - | U | Y | U | U | U | N | N | Y | H | High |
| Sittig 2016 | Y | Y | N | H | U | - | U | Y | Y | L | N | N | N | N | H | High |
| Bousema 2016 | Y | Y | Y | L | U | - | U | N | U | H | U | N | N | N | H | High |
| Pierce 2010 | Y | N | N | H | N | N | H | U | Y | U | U | Y | N | N | H | High |
| Valvano 2009 | Y | Y | N | H | U | - | U | U | Y | U | U | Y | Y | N | H | High |
| Chang 2005 | N | Y | N | H | U | N | H | U | U | U | U | Y | Y | N | H | High |
| **-AHT** | | | | | | | | | | | | | | | | |
| Hymel 2019 | Y | Y | N | U | N | N | H | U | U | U | Y | Y | Y | Y | L | High |
| Pfeiffer 2018 | Y | Y | N | U | Y | - | L | Y | U | U | U | Y | N | Y | U | Unclear |
| Palifka 2016 | N | N | N | H | U | - | U | U | N | H | U | N | N | N | H | High |
| Cowley 2015 | Y | Y | N | H | U | N | H | U | U | U | U | U | N | N | H | High |
| Acker 2014 | Y | Y | Y | L | N | N | H | Y | U | U | U | N | N | N | H | High |
| Hymel 2014 | Y | Y | N | H | Y | - | L | U | N | H | U | Y | Y | Y | L | High |
| Hymel 2013 | Y | Y | N | H | N | Y | H | U | U | U | U | Y | Y | Y | U | High |
| Vinchon 2009 | N | Y | N | H | U | - | U | U | U | U | U | Y | N | U | H | High |
| Vinchon 2005 | U | Y | N | H | U | Y | U | Y | U | U | U | N | N | U | H | High |
| Hettler 2003 | Y | Y | N | H | U | - | U | U | U | U | Y | Y | Y | N | H | High |
| Wells 2002 | Y | Y | Y | L | Y | N | H | U | Y | U | U | Y | Y | U | U | High |
| N=no; Y=yes; “-” =Not applicable  Questions in the critical appraisal form: 1=Was a consecutive or random sample of patients enrolled?; 2=Was a case-control design avoided?; 3=Did the study avoid inappropriate exclusions?; 4=Were the index test results interpreted without knowledge of the results of the reference standard?; 5=If a threshold was used, was it pre-specified?; 6=Is the reference standard likely to correctly classify the target condition?; 7=Were the reference standard results interpreted without knowledge of the results of the index test?; 8=Was there an appropriate interval between index test(s) and reference standard?; 9=Did all patients receive a reference standard?; 10=Did patients receive the same reference standard?; 11=Were all patients included in the analysis? | | | | | | | | | | | | | | | | |
