# Supplementary material for: Identifying children exposed to maltreatment: a systematic review update
Source: BMC Pediatr. 2020 Mar 7;20:113. doi: 10.1186/s12887-020-2015-4 (PMC7060650; doi:10.1186/s12887-020-2015-4)
Supplement: Supplementary file 5 — Additional file 5. Consequences of screening per 100,000 children [file 12887_2020_2015_MOESM5_ESM.docx]

**SUPPLEMENTAL FILE 5 - Consequences of screening depending on use of screening tool per 100,000 children**

**Study without Serious Verification or Incorporation Bias**

*SPUTOVAMO checklist:*

|  | **SPUTOVAMO Physical abuse (PA)** (1) | | **SPUTOVAMO Neglect (N)** (1) | | **Consequences of screening**** |
| --- | --- | --- | --- | --- | --- |
| **Sensitivity (%)** | 100% | | 83% | |  |
| **Specificity (%)** | 86% | | 87% | |  |
| **Prevalence*** | **2%** | **10%** | **2%** | **10%** |  |
| **True positives** | 2000 | 10,000 | 1666 | 8330 | - **SPUTOVAMO PA:** 340 to 1700 of reported suspected physical abuse cases will be substantiated; some form of post-investigation services will be received by 211 to 1054 of children with substantiated physical abuse - **SUPTOVAMO N:** 283 to 1416 of reported suspected neglect cases will be substantiated; some form of post-investigation services will be received by 176 to 878 of children with substantiated neglect |
| **False negatives**  **(missed cases)** | 0 | 0 | 334 | 1670 | - **SPUTOVAMO PA:** 0 children will be missed - **SPUTOVAMO N:** 84 to 418 will not receive access to the mental health services they need |
| **True negatives** | 84,770 | 77,850 | 84,966 | 78,030 | Children will appropriately not be labelled as being potentially maltreated and will not undergo an investigation or be treated for maltreatment that they have not been exposed to. |
| **False positives**  **(over treated)** | 13,230 | 12,150 | 13,034 | 11,970 | - **SPUTOVAMO**-**PA:** 12,150 to 13, 230 could receive a distressing CPS investigation - **SPUTOVAMO-N:** 11,970 to 13, 034 could receive a distressing CPS investigation |
| Physical abuse (PA), Neglect (N)  *Prevalence rates of 2% to 10% children exposed to maltreatment presenting to emergency rooms (2).  ******According to data from the United States, 17% of children who are reported to child welfare are considered to have substantiated maltreatment, 1% indicated, and 82% are classified as non-victims (3). Of the children with substantiated maltreatment, 62% may receive post investigation services. In addition, 25-50% of children exposed to maltreatment need treatment for mental health concerns (calculations in table based on 25%) (4). | | | | | |

**Studies with Serious Verification or Incorporation Bias**

*SPUTOVAMO checklist:*

|  | **SPUTOVAMO Child Maltreatment (CM)** (5) | | **SPUTOVAMO CM** (6) | | **Consequences of screening**** |
| --- | --- | --- | --- | --- | --- |
| **Sensitivity (%)** | 78% | | 15% | |  |
| **Specificity (%)** | 36% | | 98% | |  |
| **Prevalence*** | **2%** | **10%** | **2%** | **10%** |  |
| **True positives** | 1564 | 7821 | 295 | 1475 | Per 100,000 children suspected of maltreatment who are reported to CPS:   - **SPUTOVAMO-CM** (5)**:** 266 to 1330 of reported suspected maltreatment cases will be substantiated; some form of post-investigation services will be received by 165 to 824 of children with substantiated maltreatment - **SPUTOVAMO-CM** (6)**:** 50 to 251 of reported suspected maltreatment cases will be substantiated; some form of post-investigation services will be received by 31 to 155 of children with substantiated maltreatment |
| **False negatives**  **(missed cases)** | 436 | 2179 | 1705 | 8525 | Per 100,000 children who are missed:   - **SPUTOVAMO-CM** (5)**:** 109 to 545 will not receive access to the mental health services they need - **SPUTOVAMO-CM** (6): 426 to 2131 will not receive access to the mental health services they need |
| **True negatives** | 34,939 | 32,087 | 96,246 | 88,389 | Children will appropriately not be labelled as being potentially maltreated and will not undergo an investigation or be treated for maltreatment that they have not been exposed to. |
| **False positives**  **(over treated)** | 63,061 | 57,913 | 1754 | 1611 | Per 100,000 children who are over treated:   - **SPUTOVAMO-CM** (5)**:** 57,913 to 63,061 could receive a distressing CPS investigation - **SPUTOVAMO-CM** (6): 1611 to 1754 could receive a distressing CPS investigation |
| Child maltreatment (CM), Physical abuse (PA)  *Prevalence rates of 2% to 10% children exposed to maltreatment presenting to emergency rooms (2).  ******According to data from the United States, 17% of children who are reported to child welfare are considered to have substantiated maltreatment, 1% indicated, and 82% are classified as non-victims (3). Of the children with substantiated maltreatment, 62% may receive post investigation services. In addition, 25-50% of children exposed to maltreatment need treatment for mental health concerns (calculations in table based on 25%) (4). | | | | | |

***Top-to-toe physical inspection:***

|  | **Top-to-toe physical inspection (CM)** (5) | | **Consequences of screening**** |
| --- | --- | --- | --- |
| **Sensitivity (%)** | 53% | |  |
| **Specificity (%)** | 54% | |  |
| **Prevalence*** | **2%** | **10%** |  |
| **True positives** | 1057 | 5283 | Per 100,000 children suspected of maltreatment who are reported to CPS:   - **Top-to-toe physical inspection:** 180 to 898 of reported suspected maltreatment cases will be substantiated; some form of post-investigation services will be received by 111 to 557 of children with substantiated maltreatment |
| **False negatives**  **(missed cases)** | 943 | 4717 | Per 100,000 children who are missed:   - **Top-to-toe physical inspection:** 236 to 1179 will not receive access to the mental health services they need |
| **True negatives** | 52,769 | 48,462 | Children will appropriately not be labelled as being potentially maltreated and will not undergo an investigation or be treated for maltreatment that they have not been exposed to. |
| **False positives**  **(over treated)** | 45,231 | 41,538 | Per 100,000 children who are over treated:   - **Top-to-toe physical inspection:** 41,538 to 45,231 could receive a distressing CPS investigation |
| Child maltreatment (CM)  *Prevalence rates of 2% to 10% children exposed to maltreatment presenting to emergency rooms (2).  ******According to data from the United States, 17% of children who are reported to child welfare are considered to have substantiated maltreatment, 1% indicated, and 82% are classified as non-victims (3). Of the children with substantiated maltreatment, 62% may receive post investigation services. In addition, 25-50% of children exposed to maltreatment need treatment for mental health concerns (calculations in table based on 25%) (4). | | | |

***Escape instrument:***

|  | **Escape CM (1)** (7) | | **Escape CM (2)** (8) | | **Consequences of screening**** |
| --- | --- | --- | --- | --- | --- |
| **Sensitivity (%)** | 80% | | 100% | |  |
| **Specificity (%)** | 98% | | 98% | |  |
| **Prevalence*** | **2%** | **10%** | **2%** | **10%** |  |
| **True positives** | 1600 | 8000 | 2000 | 10,000 | Per 100,000 children suspected of maltreatment who are reported to CPS:   - **Escape CM (1):** 272 to 1360 of reported suspected maltreatment cases will be substantiated; some form of post-investigation services will be received by 169 to 843 of children with substantiated maltreatment - **Escape CM (2):** 340 to 1700 of reported suspected maltreatment cases will be substantiated; some form of post-investigation services will be received by 211 to 1054 of children with substantiated maltreatment |
| **False negatives**  **(missed cases)** | 400 | 2000 | 0 | 0 | Per 100,000 children who are missed:   - **Escape-CM (1):** 100 to 500 will not receive access to the mental health services they need - **Escape-CM (2):** 0 children are missed |
| **True negatives** | 95,978 | 88,143 | 96,357 | 88,491 | Children will appropriately not be labelled as being potentially maltreated and will not undergo an investigation or be treated for maltreatment that they have not been exposed to. |
| **False positives**  **(over treated)** | 2022 | 1857 | 1643 | 1509 | Per 100,000 children who are over treated:   - **Escape-CM (1):** 1857 to 2022 could receive a distressing CPS investigation - **Escape**-**CM (2):** 1509 to 1643 could receive a distressing CPS investigation |
| Child maltreatment (CM)  *Prevalence rates of 2% to 10% children exposed to maltreatment presenting to emergency rooms (2).  ******According to data from the United States, 17% of children who are reported to child welfare are considered to have substantiated maltreatment, 1% indicated, and 82% are classified as non-victims (3). Of the children with substantiated maltreatment, 62% may receive post investigation services. In addition, 25-50% of children exposed to maltreatment need treatment for mental health concerns (calculations in table based on 25%) (4). | | | | | |

References

1. Sittig JS, Uiterwaal CSPM, Moons KGM, Russel IMB, Nievelstein RAJ, Nieuwenhuis EES, et al. Value of systematic detection of physical child abuse at emergency rooms: a cross-sectional diagnostic accuracy study. BMJ Open. 2016 Mar 1;6(3):e010788.

2. Louwers ECFM, Affourtit MJ, Moll HA, de Koning HJ, Korfage IJ. Screening for child abuse at emergency departments: a systematic review. Arch Dis Child. 2010 Mar;95(3):214–8.

3. U.S. Department of Health & Human Services, Administration for Children and Families, Administration on Children, Youth and Families, Children’s Bureau. Child maltreatment 2015 [Internet]. 2017. Available from: http://www.acf.hhs.gov/programs/cb/research-data-technology/statistics-research/child-maltreatment.

4. Wang C-T, Holton J. Total estimated cost of child abuse and neglect in the United States [Internet]. Chicago, Illinois: Prevent Child Abuse America; 2007 Sep. Available from: http://icctc.org/PMM%20Handouts/PCAACostAnalysisCAN2007.pdf

5. Teeuw AH, Kraan RBJ, van Rijn RR, Bossuyt PMM, Heymans HSA. Screening for child abuse using a checklist and physical examinations in the emergency department led to the detection of more cases. Acta Paediatr Oslo Nor 1992. 2018 Jul 10;

6. Schouten MC, Stel HF van, Verheij TJ, Houben ML, Russel IM, Nieuwenhuis EE, et al. The value of a checklist for child abuse in out-of-hours primary care: To screen or not to screen. PLOS ONE. 2017 Jan 3;12(1):e0165641.

7. Louwers ECFM, Korfage IJ, Affourtit MJ, Ruige M, van den Elzen APM, de Koning HJ, et al. Accuracy of a screening instrument to identify potential child abuse in emergency departments. Child Abuse Negl. 2014 Jul;38(7):1275–81.

8. Dinpanah H, Pasha AA, Sanji M. Potential child abuse screening in emergency department: a diagnostic accuracy study. Emergency. 2016 Dec 26;5(1):8.
